# Induced Expression of *Xerophyta viscosa XvSap1* Gene Enhances Drought Tolerance in Transgenic Sweet Potato

**DOI:** 10.3389/fpls.2019.01119

**Published:** 2019-09-20

**Authors:** Wilton Mbinda, Christina Dixelius, Richard Oduor

**Affiliations:** ^1^Department of Biochemistry and Biotechnology, Pwani University, Kilifi, Kenya; ^2^Swedish University of Agricultural Sciences, Department of Plant Biology, Uppsala BioCenter, Linnean Center for Plant Biology, Uppsala, Sweden; ^3^Department of Biochemistry, Microbiology and Biotechnology, Kenyatta University, Nairobi, Kenya

**Keywords:** drought stress, gene expression, sweet potato, *Xerophyta viscosa*, *XvSap1* gene

## Abstract

Drought stress often leads to reduced yields and is a perilous delimiter for expanded cultivation and increased productivity of sweet potato. Cell wall stabilization proteins have been identified to play a pivotal role in mechanical stabilization during desiccation stress mitigation in plants. They are involved in numerous cellular processes that modify cell wall properties to tolerate the mechanical stress during dehydration. This provides a plausible approach to engineer crops for enhanced stable yields under adverse climatic conditions. In this study, we genetically engineered sweet potato cv. Jewel with *XvSap1* gene encoding a protein related to cell wall stabilization, isolated from the resurrection plant *Xerophyta viscosa*, under stress-inducible XvPSap1 promoter *via Agrobacterium*-mediated transformation. Detection of the transgene by PCR, Southern blot, and quantitative real-time PCR (qRT-PCR) analyses revealed the integration of *XvSap1* in the three independent events. Phenotypic evaluation of shoot length, number of leaves, and yield revealed that the transgenic plants grew better than the wild-type plants under drought stress. Assessment of biochemical indices during drought stress showed higher levels of chlorophyll, free proline, and relative water content and decreased lipid peroxidation in transgenic plants than in wild types. Our findings demonstrate that *XvSap1* enhances drought tolerance in transgenic sweet potato without causing deleterious phenotypic and yield changes. The transgenic drought-tolerant sweet potato lines provide a valuable resource as a drought-tolerant crop on arid lands of the world.

## Introduction

Plant survival under adverse environmental conditions depends on integration of stress-adaptive physiological and metabolic changes into their endogenous developmental systems. The world’s seventh most important crop, sweet potato [*Ipomoea batatas* (L.) Lam.], plays a significant role in food security and nutritional requirements, for millions of people in Asia and Africa (Bovell, 2007). This crop also has an enormous potential to be commercially exploited as an industrial raw material (Kasran et al., 2015). Sweet potato is widely cultivated on marginal lands due to its relatively high tolerance to abiotic stress. The orange-fleshed sweet potato cultivars contain remarkable quantities of β-carotene, which is a viable solution to combat vitamin A deficiency in sub-Saharan Africa (Laurie et al., 2018). The crop’s effective clonal propagation, cultivation simplicity, and high biomass generation make sweet potato conceivably appropriate for molecular farming of valuable and novel products (Rukundo et al., 2017). Like other crops, sweet potato is adversely affected by drought stress, which seriously hampers crop productivity and negatively influences the expansion of sweet potato cultivation (Jin et al., 2017). Additionally, as a source of bioenergy, sweet potato will mainly be grown on marginal land in the future, which is a potentially attractive way for bioenergy production because it preserves better land for food crops and accords poor farmers a new source of income. Improving drought tolerance is essential for the crop’s adaption to environmental stress and increasing productivity. Sweet potato trait improvement through conventional breeding is constrained by its laborious and time-consuming genetics. These limitations principally arise owing to the crop’s high heterozygosity, high levels of male infertility, complicated polyploidy, and production of few seeds because of its self-incompatibility, resulting to sturdy segregation of hybrid progenies and the loss of numerous valuable traits (Martin, 1965; Mwanga et al., 2017). The fast advancement in plant biotechnology has unlocked new potentials for increasing tolerance to abiotic and biotic stresses to sweet potato as well as improving its nutritional quality by identifying key genes and introducing them through genetic engineering. Additionally, genetic engineering technology has the capability of introgressing genes from incompatible plant species or other organisms, an imperative phenomenon for crop improvement. In sweet potato, little progress has been made in the generation and evaluation of transgenic events against drought tolerance.

Plant drought stress involves intricate regulatory processes that control water flux and cellular osmotic modification through the biosynthesis of osmoprotectants (Golldack et al., 2014). “Resurrection plants” survive dehydration (losing over 90% of their water content) of their vegetative tissues to air-dry state for extended periods and recover complete metabolic state after rehydration (Challabathula et al., 2016). These resurrection plants could serve as ideal models for the ultimate design of important crops with enhanced stress tolerance. A representative of this special category of plants is *Xerophyta viscosa*. This species is a Southern African native monocot species that has been extensively investigated to understand the genetic mechanisms of desiccation tolerance and may serve as potential plethora of superior genes that could introgress into important crops (Costa et al., 2017). Of particular interest in this study is *XvSap1*, a stress-regulated protein, which was isolated from a complementary DNA (cDNA) library constructed from dehydrated *X. viscosa* leaves. This gene has been associated with desiccation stress tolerance (Garwe et al., 2003). *XvSap1* has 49% identity to a cold acclimation protein, WCOR413, from *Triticum aestivum* (Mohammadi et al., 2007). Earlier works on utilizing genes from *X. viscosa* have resulted in promising outcomes on drought stress improvement in important crops such as maize (Seth et al., 2016) and tobacco (Kumar et al., 2013), which encouraged us to exploit *XvSap1* for genetic engineering of sweet potato.

The *XvSap1* gene is a highly hydrophobic protein. It encodes two membrane lipoprotein–lipid domains in which either one or both are activated in leaves of *X. viscosa* during dehydration stress (Garwe et al., 2003). Although the function of *XvSap*1 in *X. viscosa* is yet to be fully determined, there are three proposed hypotheses. First, *XvSap1* may be involved in stabilizing the plasma membrane; second, it may be involved in maintaining ion homeostasis; and last, *XvSap1* may be a G-protein-coupled receptor (GPCR) associated with signal transduction in osmotic stress (Iyer et al., 2008). GPCRs form a large family of proteins whose principal function is to transduce extracellular stimuli into intracellular signals (Kroeze et al., 2003). *Arabidopsis thaliana* and *Nicotiana tabacum* transgenic plants overexpressing *XvSap1* exhibited high tolerance to osmotic, salt, heat, and dehydration stress (Garwe et al., 2006). In the present study, we generated and assessed transgenic sweet potato plants expressing *XvSap1* under the control of its stress-inducible endogenous promoter. The results demonstrate that transgenic sweet potato plants expressing *XvSap1* had significantly improved tolerance to drought stress compared with the wild-type plants.

## Materials and Methods

### Vector Construction for Plant Transformation

The full-length cDNA of *XvSap1* (accession no. CB330588.1), as well as its stress-inducible promoter, was provided by Prof. Jennifer A. Thomson (University of Cape Town, South Africa) and was cloned into the binary vector pNOV2819 at the *Bam*HI and *Hin*dIII restriction sites. The binary vector pNOV2819 contains the phosphomannose isomerase (*pmi*) gene as a selectable marker that confers resistance to mannose. Since sweet potato is not sensitive to mannose selection (Mbinda et al., 2015), the *pmi* was substituted with the neomycin phosphotransferase (*npt*II) gene flanked by the *nos* promoter and *nos* terminator at *Hin*dIII and *Kpn*I restriction sites. The binary vector harboring the transgene (pSAP1-*XvSap1*) and the selectable marker *npt*II, conferring kanamycin resistance, was mobilized into the disarmed *Agrobacterium tumefaciens* strain EHA105 *via* the freeze–thaw method (Weigel and Glazebrook, 2006) and used for sweet potato genetic transformation.

### Sweet Potato Genetic Transformation

Sweet potato [*I. batatas* (L.) Lam., cv. Jewel, donated by the International Potato Centre, Nairobi, Kenya] plants were used in this study. The plants were maintained *in vitro* by subculturing every 4 weeks at 27 ± 1 °C under 16 h/8 h light/dark conditions (Mbinda et al., 2016). Explants were prepared from stem segments of 3- to 4-week-old *in vitro* plants by transversely cutting them into 6- to 10-mm segments before dividing the segments along the axis. Genetic transformation, somatic embryogenesis, and regeneration of putative transgenic plants were performed (Mbinda et al., 2018). Cefotaxime (250 mg/L) and kanamycin (50 mg/L) were supplemented to the medium to induce the development of transgenic cell clusters. The regenerated sweet potato plantlets were transferred to soil and grown in a greenhouse at 27 ± 1°C.

### DNA Extraction, PCR, and Southern Blot Analysis

Total genomic DNA was extracted from leaves of both wild-type and putative transgenic sweet potato lines using the cetyltrimethylammonium bromide (CTAB) method (Borges et al., 2009). The extracted genomic DNA was amplified using gene-specific primers (**Table S1**) to evaluate the presence or absence of *XvSap1*. Southern blotting was performed to evaluate the copy number and stable integration of the transgene in the transgenic sweet potato lines using standard techniques (Sambrook et al., 1989). Genomic DNA (20 µg) from PCR-positive and wild-type plant lines was digested with *Eco*RI. Agarose (0.8%) gel electrophoresis was used to separate the digested DNA, before blotting onto a Hybond-N+ membrane, followed by UV cross-linking. A probe, amplified from the *XvSap1* plasmid (**Table S2**), was labeled with deoxycytidine triphosphate (dCTP) α-^32^P using Amersham Rediprime II DNA labeling kit (GE Healthcare, Buckinghamshire, England) and used for hybridization at 42°C. Blots were washed for 10 min at 42 °C and more stringently twice for 30 min at 55°C. A third wash was done if the residual radioactivity on the membrane was considered high. Finally, the membrane was monitored using a Kodak Flexible Phosphor Imager (Eastman Kodak Co., New York, USA).

### qRT-PCR Analysis

Leaves from water-stressed and control plants were collected at 0, 3, 6, 9, and 12 days after simulated drought stress. Total RNA was isolated using a Spectrum Plant Total RNA Kit (Sigma-Aldrich, St. Louis, USA). First-strand cDNA was synthesized using gene-specific primers (**Table S3**). Quantitative real-time PCR (qRT-PCR) was performed with Bio-Rad iQ5 l System Software 1.0. The sweet potato ubiquitin gene was used as the internal control (Park et al., 2012). Relative expression values were analyzed with the comparative 2^−ΔΔCT^ formula as described by Livak and Schmittgen (2001).

### Drought Tolerance Analysis

Wild-type and transgenic sweet potato stem cuttings (12 cm) were planted into pots containing mixed soil in greenhouse under 750–1000 µmol E m^−2^ S^−1^ of photosynthetic photon flux density with 28 ± 2°C, 16/8 day photoperiod, and 80 ± 5% relative humidity for 4 weeks. Thereafter, and in a completely randomized design, two groups of plant treatments were set for 12 days: control conditions [61.7% soil water content (SWC)] and simulated water deficit stress (0.3% SWC). Morphometric parameters (shoot length and number of leaves), biochemical assays [chlorophyll, proline, malondialdehyde (MDA), and relative water contents], and the *XvSap1* expression analysis of the different materials were evaluated. Three biological replications were prepared for each treatment. Further, transgenic and wild-type sweet potato plants were planted in pots and regularly irrigated for 30 days. Thereafter, the plants were exposed to drought stress for 90 days. Yield attributes in terms of fresh weight (FW) and dry matter were measured for comparative analysis. Control plants (wild-type and transgenic plant lines) were irrigated throughout the experiment.

### Assessment of SWC

Soil samples collected at 0, 3, 6, 9, and 12 days after withholding water were dried at 105°C. The SWC, as a measure of “drought” severity was calculated using the weight fraction described by Coombs et al. (1985): SWC (%) = [(FW − DW)/DW] × 100, where FW was the fresh weight for the soil portion from the internal area of sampled pots and DW was the dry weight for the soil portion after drying at 105°C for 4 days.

### Measurement of Photosynthetic Pigment

Chlorophyll contents, under control and drought conditions, were measured in attached leaves of both wild-type and transgenic plant lines with, SPAD chlorophyll meter (Minolta Co., Osaka, Japan), a nondestructive portable device. The youngest fully expanded leaf flag from the top of each plant was used. Measurement started 9 days prior to commencement of water deficit stress and continued for 12 days. The mean of the five values was taken per plant for each of the three replications.

### Estimation of Proline Content

Colorimetric assay was used to analyze free proline content. Fresh leaf tissue (100 mg) was homogenized in 10 ml of 3% (w/v) sulfosalicylic acid. The homogenate was filtered through a No. 1 Whatman filter paper. A 0.1-ml aliquot of the filtrate was mixed with a 0.5-ml reaction solution [40% (w/v) acidic ninhydrin (8.8 µM of ninhydrin, 10.5 M of glacial acetic acid, and 2.4 M and orthophosphoric acid), 40% (v/v) glacial acetic acid, and 20% (v/v) of 3% (v/v) sulfosalicylic acid]. The samples were incubated for 60 min at 100°C, and the reaction was terminated by incubating the samples on ice for 5 min. Free proline from the samples was extracted by adding 1 ml of toluene and vortexing for 20 s. The absorbance at 520 nm was measured with a UVmini-1240 ultraviolet–visible (UV-Vis) spectrophotometer (Shimadzu, Kyoto, Japan) using toluene as a reference. Proline content (µmol/g FW) in leaf tissues was computed from a standard curve using 0–100 µg of l-proline according to a formula by Bates et al. (1973).

### Analysis of Lipid Peroxidation

Lipid peroxidation was measured in terms of MDA content, a product of lipid peroxidation, which is an indicator of oxidative damage caused by stress such as drought and salinity in plants (Del Rio et al., 2005). Leaf tissue of 100 mg was homogenized by adding 0.5 ml of 0.1% (w/v) trichloroacetic acid. The homogenate was centrifuged at full speed for 10 min at 4°C. The collected supernatant was mixed with a 0.5-ml solution (1.5 ml of 0.5% thiobarbituric acid diluted in 20% trichloroacetic acid). The mixture was incubated 95 °C in water bath at for 25 min before ending the reaction by incubating on ice for 10 min. The absorbance was measured at 532 and 600 nm using a spectrophotometer (UVmini-1240 UV-Vis) with 1% thiobarbituric acid in 20% trichloroacetic acid as control. The concentration of MDA (µmol/g FW), calculated as a measure of lipid peroxidation, was determined according to Health and Packer (1968).

### Determination of Relative Water Content

Water loss in plants by transpiration was measured form a detached-leaf assay of the excised third leaf from wild-type and transgenic sweet potato plants. FW of the leaves was recorded immediately after excision and further incubated in a Petri plate at 28°C in deionized water. The weight of these leaves was recorded after 1, 2, 3, 5, and 17 h. The rate of water loss was determined according to the formula described by Turner (1981).

#### Statistical Analysis

All experiments were carried out at least in three biological replicates, measurements were done in triplicate, and data were represented as mean ± standard deviation (SD). The data collected were analyzed using one-way analysis of variance and *post hoc* Fisher’s least significant difference test to determine significant differences between the means of each treatment. A *P* value of 0.05 at a confidence level of 95% was considered to be statistically significant. The Minitab 17 statistical software was used for data analysis. All graphs were drawn using the SigmaPlot software. Data are presented in mean values ± SD.

## Results

### Sweet Potato Transformation

**Figure 1:**
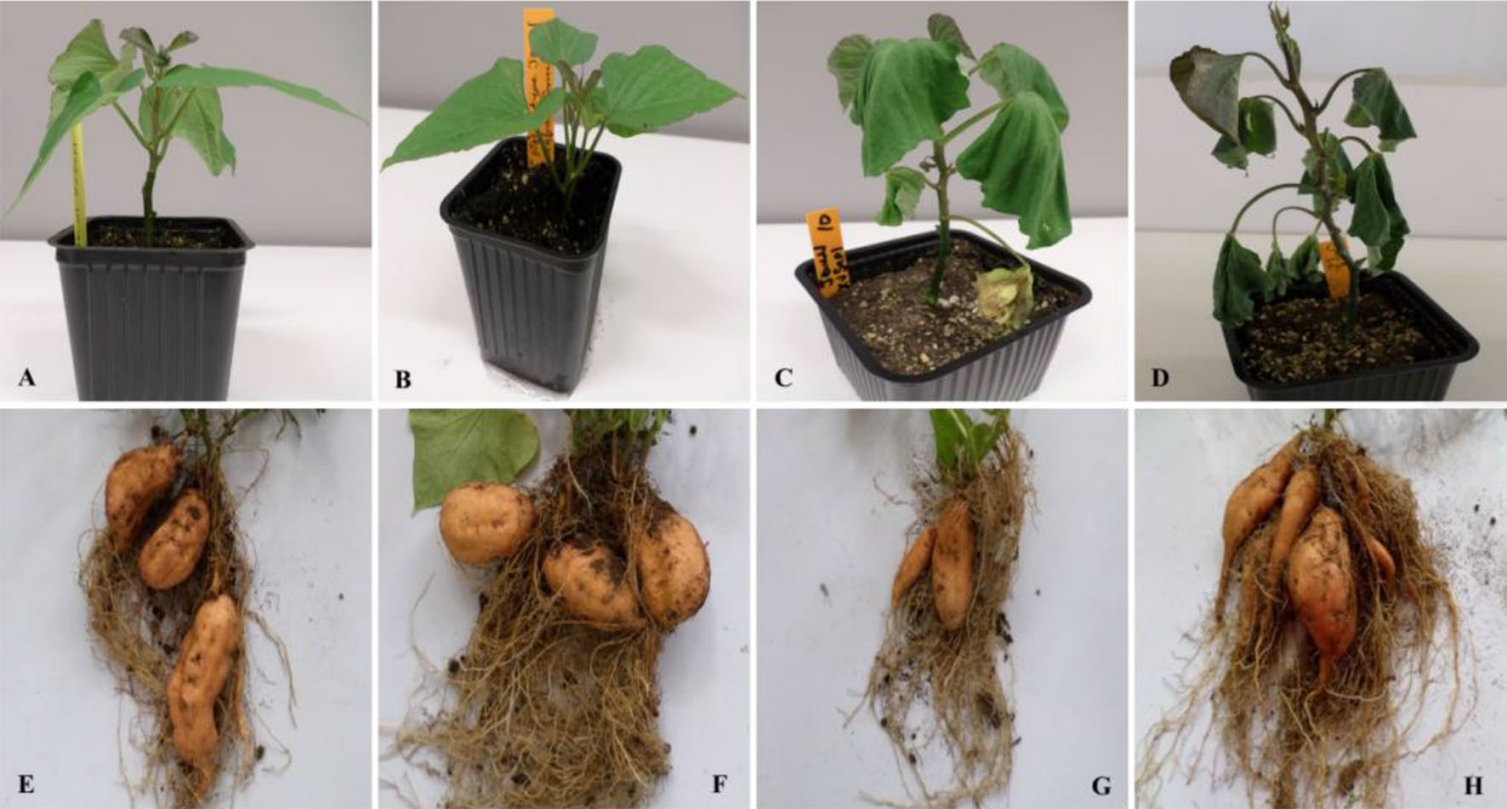
Responses to drought stress in transgenic and wild-type sweet potato plants. **(A** and **B)** Morphological appearance of transgenic and wild-type plants, respectively. **(C** and **D)** Phenotypes of transgenic and wild-type plants grown in soil after 12 days of drought stress, respectively. **(E** and **F)** Yield performance of wild-type and transgenic plants grown under control conditions, respectively. **(G** and **H)** Yield performance of wild-type and transgenic plants grown under drought conditions for 90 days, respectively.

A total of 167 sweet potato stem explants were cocultivated with *Agrobacterium* strain EHA101 carrying pNOV2819-*XvSap1* binary vector (**Figure S1**). The transformed stem segments gave rise to 133 kanamycin-resistant calli, resulting to a transformation efficiency of 79.6%. Three putative transgenic sweet potato plants were regenerated from 117 surviving resistant calli. The regenerated sweet potato plantlets were hardened for propagation in a biosafe greenhouse. Under control conditions (25–27°C and 61.7% SWC), all transgenic sweet potato lines were phenotypically and morphologically indistinguishable from the wild-type lines (**Figures 1A, B**).

### Molecular Characterization of Transgenic Sweet Potato Plants

**Figure 2:**
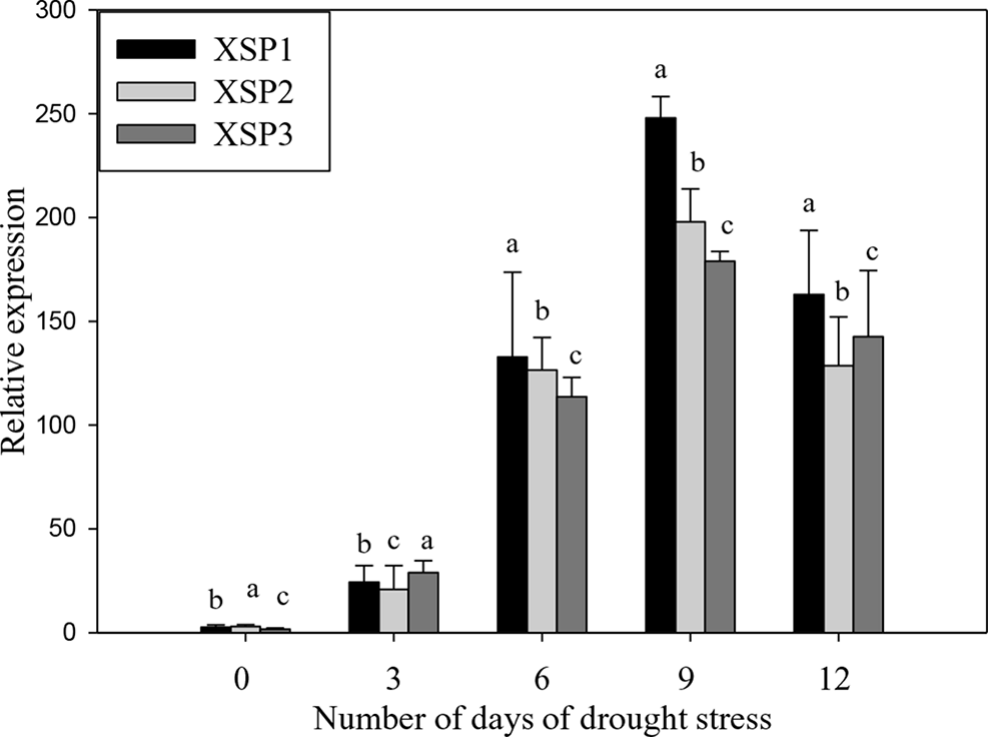
Quantitative real-time PCR (qRT-PCR) analysis of *XvSap1* transcript levels in leaves of transgenic sweet potato XSP1, XSP2, and XSP3 transgenic lines during 12-day water deficit stress. Means ± SE (standard error) followed by different alphabets in each growth condition are significantly different *(P ≤ 0.05)* using Fisher’s least significant difference (LSD). Data are from three independent replicates of the same event (**Table S4**).

PCR analysis showed the presence of the 464-bp *XvSap1* fragment in the transformed plants, but this was absent in the nontransformed wild-type plants (**Figure S2**). The results of Southern hybridization assay clearly showed that all transgenic lines (XSP1, XSP1, and XSP3) contained a single-copy insertion (**Figure S3**). Further, results of reverse-transcription PCR (RT-PCR) analysis of the three transgenic independent events showed *XvSap1* mRNA transcripts, which were absent in the nontransformed control plants (**Figure S4**). Further, we investigated the transcript expression levels in leaves of wild-type and transgenic lines under control and drought stress conditions by qRT-PCR. The expression levels of *XvSap1* in the three transgenic plants under the control condition were low but were induced under stress to 50-fold after 6 days of withholding water. Thereafter, expression started to decline, which is expected when using a stress-induced promoter (**Figure 2**).

### Phenotypic Responses to Water Stress

**Figure 3:**
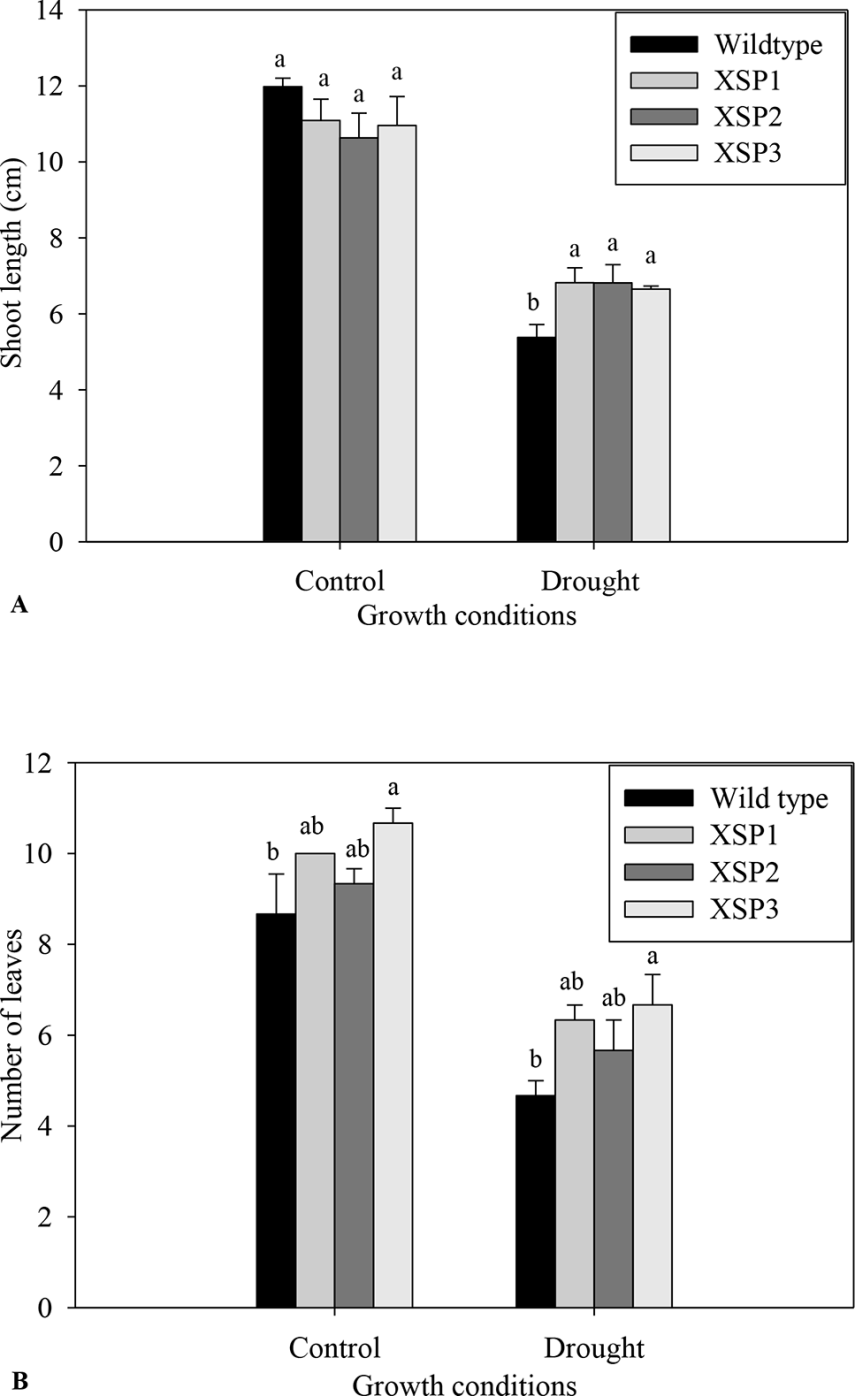
Phenotypic index changes of wild-type and *XvSap1* transgenic sweet potato plants under control and drought stress conditions. **(A)** Shoot length. **(B)** Number of leaves. Means ± SE (standard error) followed by different alphabets in each growth condition are significantly different (*P ≤ 0.05*) using Fisher’s least significant difference (LSD). Data are from three independent replicates of the same event. (**Table S5** and **Table S6**).

Under control conditions, 4-week-old transgenic and nontransformed control sweet potato plant lines did not have visible phenotypic differences (**Figures 1A, B**). Before the water deficit stress experiments, the different sweet potato plant types had no significant difference in shoot length. Upon exposure of the plants to severe water deficit stress (0.3% SWC), the shoot length of wild-type control plants reduced by about 55.%, whereas the transgenic plant shoot length decreased by a lower margin of 37.8% (**Figure 3A**). Similarly, after drought stress treatment, the average number of leaves in transgenic sweet potato plants slightly declined by an average of 37.8% as compared with the wild-type plants, which had a drastic decline of 44.3% from an average of 8.7 leaves to 4.7 leaves (**Figure 3B**). After 6 days of moderate water deficit stress (27.8% SWC), severe wilting was evidently observed in the leaves of the untransformed plants (**Figure 1D**), but the leaves of transgenic plants showed only minor damage (**Figure 1C**). Severe dehydration symptoms were observed in the transgenic plants following 12 days of water deficit stress (0.3% SWC). The severe symptoms included wilted leaves, drying up of leaf edge and tip, and brown leaves. In both the wild-type and transgenic plant lines, older leaves evidently displayed leaf wilting and chlorosis when the plants were exposed to severe drought stress. Overall, the nontransformed plants displayed much more severe symptoms compared with the transgenic plants.

**Figure 4:**
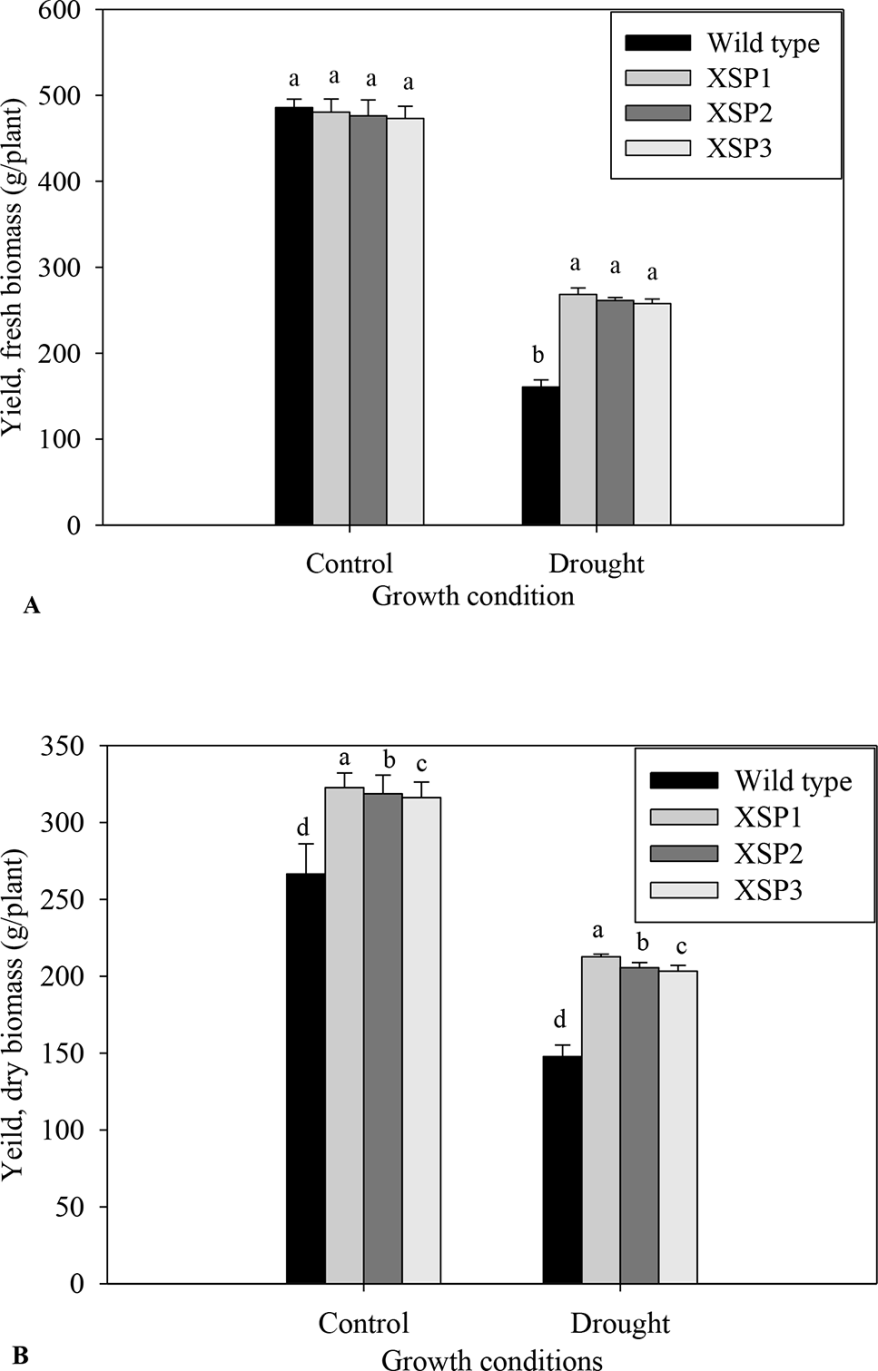
Yield attributes of tolerant transgenic plants and wild-type plants grown in the drought stress. **(A)** Fresh weight. **(B)**. Dry matter. Means ± SE (standard error) followed by different alphabets in each growth condition are significantly different (*P ≤ 0.05*) using Fisher’s least significant difference (LSD). Data are from three independent replicates of the same event (**Table S7** and **Table S8**).

Under control conditions, there was no phenotypic difference in the tuber formation between the untransformed (**Figure 1E**) and transgenic plants (**Figure 1F**). Following 90 days of drought stress, the yield of wild-type plants (**Figure 1G**) drastically reduced compared with transgenic plants that produced bigger and higher numbers of tubers (**Figure 1H**, **Table S9**). When plants were regularly watered, the biomass of fresh yield and dry yield of both wild-type and transgenic plants did not differ (**Figures 4A, B**).

To evaluate the effect of *XvSap1* in transgenic sweet potato, the root biomass was measured upon subjecting the plants to drought stress for 90 days. We found that both the FW and DW of transgenic and wild-type plants were significantly different in drought stress and the transgenic sweet potato lines displayed higher root dry biomass compared with the wild-type plants (**Figures 4A, B**). Similarly, transgenic sweet potato lines had more tubers than the wild-type plants under drought stress (**Table S8**). These results indicate that inducible expression of *XvSap1* in sweet potato might promote root initiation and elongation. The average FW and DW yields per plant of transgenic plants under drought conditions were about 262.5 and 207.2 g, respectively, whereas wild-type plants recorded 160.74 and 147.74 g, respectively (**Figures 4A, B**).

### Physiological Characteristics

#### Chlorophyll Content Estimation

**Figure 5:**
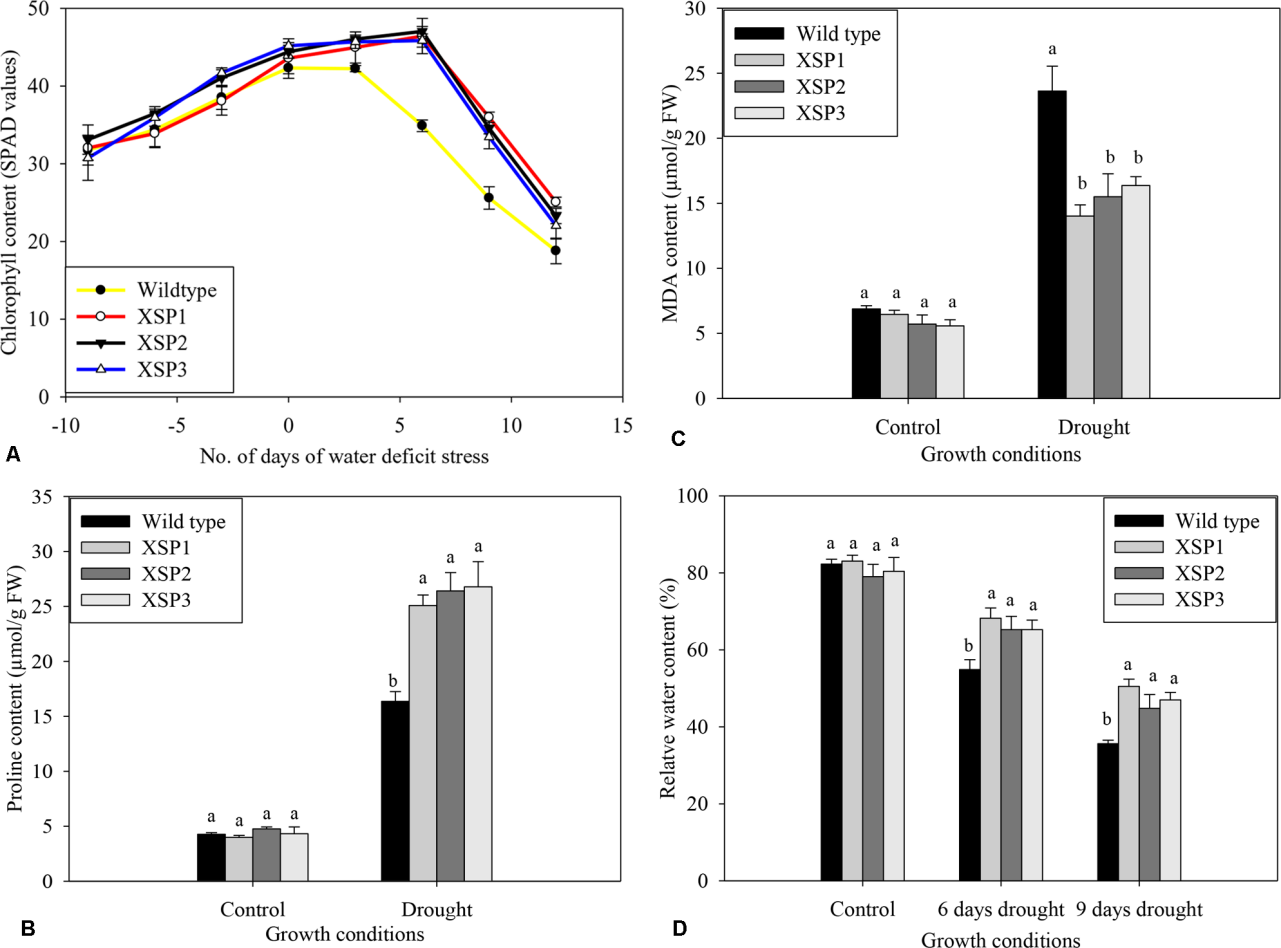
Biochemical index changes of wild type and *XvSap1* transgenic sweet potato plants under control and drought stress conditions. **(A)** Chlorophyll content. **(B)** Proline content. **(C)** Malondialdehyde content. **(D)** Water content. Means ± SE (standard error) followed by different alphabets in each growth condition are significantly different (*P ≤ 0.05*) using Fisher’s least significant difference (LSD). Data are from three independent replicates of the same event (**Table S10−S13**).

At the start of the experiment, chlorophyll contents of both wild-type and transgenic sweet potato lines were the same and increased as the plants grew (**Figure 5A**). Chlorophyll concentration in leaves of both wild-type and transgenic plant lines progressively dropped with increasing drought stress intensity, resulting in lower SPAD values compared with control treatment; plants were grown under control conditions throughout the experiment. In contrast, water deficit stress had a mild effect on chlorophyll content in leaves of all the transgenic lines compared with control, as shown in **Figure 5A**.

#### Phenotypic Change in Proline Content

To evaluate the changes brought about by drought stress, we measured the accumulation of free proline in the shoot leaves of the nontransformed and the transgenic lines, because the level of free proline is known to be an indicator of drought stress resistance capacity in plants (Zandalinas et al., 2018). No significant difference in free proline concentration was observed in both wild-type and transgenic leaves under control conditions (**Figure 5B**). After 12 days of water deficit stress experiments, the proline content in wild-type plants increased 3.8-fold to 16.4 µg/g FW (**Figure 5B**). In contrast, the transgenic lines exhibited significantly higher accumulation of proline ranging from 5.5- to 6.2-fold change increase under drought conditions (**Figure 5B**).

#### Lipid Peroxidation

Further validation for the effect of *XvSap1* expression on lipid peroxidation was carried out by estimation of MDA in the leaves. Under control conditions, the levels found in the wild-type line and the XSP1, XSP3, and XSP3 transgenic sweet potato plant lines were similar (**Figure 5C**). In contrast, imposition of water deficit stress to the plants resulted in about 3.4-fold increase in the MDA content in leaves of the wild-type plant line and much less MDA content in leaves of the transgenic sweet potato plants (**Figure 5C**).

#### Response to Relative Water Content

Under control conditions, there were no significant differences in the leaf relative water content between control and transgenic sweet potato plant XSP1 lines, and the relative water content of both lines was about 82% (**Figure 5D**). After subjecting the plants to drought stress for 6 days, the relative water content of the control wild-type leaves rapidly reduced by 37.0% to 51.8%, while the average relative water content of the transgenic lines declined from 80.8% to 66.2%, 14.6% decline (**Figure 5D**). After 12 days of water deficit stress, the leaf relative water content of the transgenic plant had decreased by just 38.4% as compared with 56.7% in the wild-type plants (**Figure 5D**). These results demonstrate that the untransformed sweet potato line was more drought sensitive than the transgenic lines.

## Discussion

As drought frequency and severity are projected to increase in the future due to climate change, the ensuing deficit in water, especially in arid and semiarid regions, will greatly reduce agronomic productivity. Increasing tolerance to drought stress through the development of crop varieties with improved tolerance is the only probable solution to extremely reduce the impacts of drought stress on important crops such as sweet potato. Through several studies, various stress tolerance genes that have profound effects on enhancing drought tolerance in plants have been identified, and among them are the members of GPCR genes. GPCRs play a crucial role in regulating plant water status under normal and adverse circumstances (Bai et al., 2018; Lu et al., 2018; Wong et al., 2018).

Numerous strategies have been applied to engineer plants with enhanced stress tolerance with limited success. One major problem is due to the undesirable or unfavorable phenotypic changes such as growth retardation and decrease in yield. Compared with wild-type plants, transgenic sweet potato plants had no observable phenotypic changes. Similar results were also observed in our previous study when sweet potato was genetically engineered with an aldose reductase, *XvAld1*, isolated from *X. viscosa* (Mbinda et al., 2018). Other studies using genes from *X. viscosa* and transferred into other crops, such as maize (Seth et al., 2016) and tobacco (Kumar et al., 2013), have also been successful, reaffirming the proposition that *X. viscosa* has prodigious potential for mining of valuable genes for biotechnological utilization with no nocuous variations to the accruing transgenic plants.

Under water deficit stress conditions, transgenic sweet potato plants were identified as drought tolerant based on the reduction proportion of vine length and number of leaves compared with untransformed plants. Upon rewatering after induced water deficit stress treatment, transgenic plants resulted in rapid recovery with higher shoot height. Results from previous work corroborate our finding that dehydration stress in plant causes hydraulic restriction, with the ensuing high tension in the xylem water column and stomatal closure (Powell et al., 2017). Expression of exogenous genes in plants has previously resulted in plant deformities. However, under nonstress conditions, we did not see any phenotypic effects of *XvSap1* expression in sweet potato. Our results therefore indicate that the introgression of *XvSap1* into sweet potato did not lead to any major observable effects in the plant architecture and growth properties. Previous similar work with genes from *X. viscosa* yielded comparable results on transgenic maize (Seth et al., 2016) and tobacco (Kumar et al., 2013), signifying that *X. viscosa* is an ideal model for the ultimate bioengineering of crops for drought tolerance enhancement.

Plant root physiological and morphological features were strongly associated with the above-ground plant organs’ growth and development and also yield formation. Active and healthy roots provide adequate nutrients, plant hormones, and water for the growth and development of plant parts above ground, which consequently promote biological yield output. On the other hand, active and health plant parts above ground provide adequate carbohydrates, which are transported to the roots and bolster the root function activities (McCormack et al., 2015). When compared with other plants, the sweet potato root structure has a characteristic that not only is the organ for nutrient absorption but also acts as a photoassimilate storage organ (Fan et al., 2012; McCormack et al., 2015). Results from our study show a remarkable improvement in both total biomass and number of tubers in transgenic lines compared with wild-type sweet potato under drought stress. This observation could be explained by the robust and better growth of the above-ground plant parts witnessed in the transgenic lines as opposed to the wild types. Our observation also established that the performance including a better-developed tuber and root system of transgenic sweet potato lines was strongly correlated with expression levels of *XvSap1*. A well and fully developed and active root system in many plants is a crucial trait for drought tolerance (Puértolas et al., 2014).

In plants, suitable amounts of reactive oxygen species (ROS) aggregation are vital for tolerance to abiotic stress (Das and Roychoudhury, 2014). Nevertheless, excess ROS generation causes degradation of chlorophyll (Dietz et al., 2016). The degradation of photosynthetic pigments, which are vital to plants principally for light harvesting during the photosynthesis process and production of reducing power, is one of the most sensitive indexes and diagnostic tools for the evaluation of differential responses to drought stress (Rolando et al., 2015). Plants can overcome drought stress by increasing the rate of chlorophyll biosynthesis which protects them by scavenging the excessive energy through thermal dissipation (Reddy et al., 2004). A decline in leaf chlorophyll concentration in response to drought stress is a common phenomenon in plants, and it is occasioned by disordering chlorophyll synthesis which results to changes in thylakoid membrane structure and eventually plant chlorosis (Kalaji et al., 2016). Our study observed that the delayed onset of induced drought in transgenic plants had advantageous consequences for their survival following induced water deficit stress. However, after long exposure to drought stress, all transgenic plants also exhibited wilting symptoms which were comparable with the symptoms developed in the untransformed sweet potato plants 7 days after water deficit stress imposition. The enhanced chlorophyll content exhibited by the transgenic lines could be a result of stabilized antioxidant status in leaf tissue drought stress. Accumulation of proline and polyamines is a common response to various abiotic stresses, and results in our study corroborate the responses of other crops to water deficit, such as kiwi fruit (Chartzoulakis et al., 1993) and cotton (Massacci et al., 2008), whose leaf chlorophyll content was higher in plants grown in control conditions than in those grown under drought conditions.

Desiccation stress induces osmotic and oxidative stress in plants, leading to cellular adaptive responses such as accumulation of compatible solutes (Chen and Murata, 2011). Free proline is the most important osmolyte and signaling molecule which accumulates generally in the cytoplasm without prejudicing normal cellular physiological functions. The amino acid also contributes in the protection of membranes, proteins, and enzymes against various stresses including drought stress (Golldack et al., 2014). Differential accumulation of free proline is therefore a common phenomenon in plant response to water deficit stress, and free proline accumulation has been reported to contribute approximately 10–15% of the osmotic adjustments in water-deficit-stressed castor plants (Babita et al., 2010), pea cultivars (Sánchez et al., 1998), and finger millet (Mukami et al., 2019). In our study, we also found the elevated levels of free proline in all transgenic sweet potato plant lines compared with the untransformed plants under water deficit stress, suggesting that *XvSap1* could induce synthesis of proline genes to confer drought tolerance in sweet potato. Our findings are consistent with the results obtained from overexpressing TsVP and BetA in maize (Wei et al., 2011), G6PDH in tobacco plants (Silva et al., 2018), and BrCIPK1 in rice (Abdula et al., 2016). The biochemical relation between the action of the *XvSap1* gene and the high proline concentration in the transgenic sweet potato plants seems to be a sensitive balance.

Further, enhanced ROS activity eventually leads to lipid peroxidation of the cell membranes and subsequent membrane leakage. This is a common phenomenon during water deficit stress and causes severe damage to plant cells, leading to programmed cell death. MDA is a product of lipid peroxidation, and its quantification is generally employed as an index of oxidative damages in plant tissues. Indeed, *XvSap1* transgenic sweet potato plants displayed less accumulation of MDA in leaves compared with untransformed plants, demonstrating that adequately enhanced cell membrane homeostasis causes drought tolerance in transgenic sweet potato plants. These results further suggest that inducible expression of *XvSap1* gene modulates oxidative stress responses. Moreover, plant growth and development are constrained by stress factors such as scavenging and quenching of free radicals occasioned by ROS. These mechanisms are extensively regulated, and plant tolerance to drought stress is related to plants’ antioxidant scavenging ability. Enhanced levels of the antioxidant constituents impede the stress damage before it develops to lethal status (Demidchik, 2015; Mickelbart et al., 2015). The higher growth and development of transgenic sweet potato plants observed in our study could be attributed to less lipid peroxidation in the transgenic plants. We hypothesize that *XvSap1* enhanced drought tolerance by increasing stabilization of cell membranes of the desiccation-stressed transgenic sweet potato plants.

Finally, the ability to retain water during dehydration is an important strategy for plant tolerance to stress caused by drought, and its measurement is a rapid approach to estimate plant water status and provides projection levels of the cellular hydration levels after drought stress treatments (Sánchez-Rodríguez et al., 2010). Relative water content usually serves as an essential indicator as to how the plant manages its water stress condition, and it is directly correlated to SWC (Hammad and Ali, 2014). Our study showed enhanced water retention capacity in *XvSap1* transgenic sweet potato plants compared with the control plants. A decreasing transpiration rate is significant for plant survival under drought conditions. Similar findings in maize (Liu et al., 2013), wheat (Le Roux et al., 2019), and *Arabidopsis* (Shao et al., 2019) have also shown that overexpression of exogenous genes improves drought tolerance by maintaining cellular full turgor during water deficit stress conditions.

In conclusion, our study results presented here provide clear evidence of the feasibility of genetically engineering sweet potato for improved drought tolerance through the inducible expression of the *XvSap1* gene, isolated from *X. viscosa*. The transgenic sweet potato lines had favorable traits associated with drought tolerance without compromising on the plant morphology, physiology, and, more importantly, the yield biomass. We believe that *XvSap1* conferred improved drought tolerance thorough cell wall mechanical stabilization during dehydration. From these results, we therefore believe that the *XvSap1* gene may have great potential in sweet potato crop improvements for drought stress tolerance.

## Data Availability

All datasets generated for this study are included in the manuscript/**Supplementary Files**.

## Author Contributions

RO and WM conceived and designed the experiments. WM performed all experiments. RO and CD supervised the execution of the research. WM analyzed the data. WM, CD, and RO wrote the manuscript.

## Funding

This research work was funded by the National Council for Science and Technology, Kenya (Grant No. NCST/5/003/3rdSTICALL/109).

## Conflict of Interest Statement

The authors declare that the research was conducted in the absence of any commercial or financial relationships that could be construed as a potential conflict of interest.
