# Supplementary material for: Induced Expression of Xerophyta viscosa XvSap1 Gene Enhances Drought Tolerance in Transgenic Sweet Potato
Source: Front Plant Sci. 2019 Sep 20;10:1119. doi: 10.3389/fpls.2019.01119 (PMC6764105; doi:10.3389/fpls.2019.01119)
Supplement: Supplementary file 1 [file DataSheet_1.docx]

## **Supplementary materials**


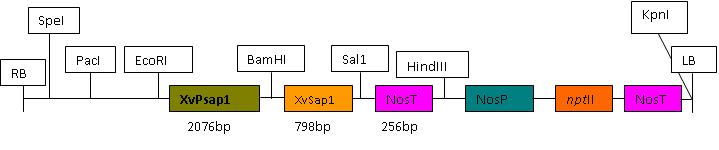


**Fig. S1** Plasmid vectors used in sweetpotato transformation. **RB,** right border of T-DNA; XvPSap1, stress inducible promoter from *X. viscosa*; *npt*II, neomycin phosphotransferase gene for plant kanamycin resistance; **NosT,** nopalin synthase terminator; XvSap1, truncated gene from *X. viscosa*; **LB,** left border of T-DNA.


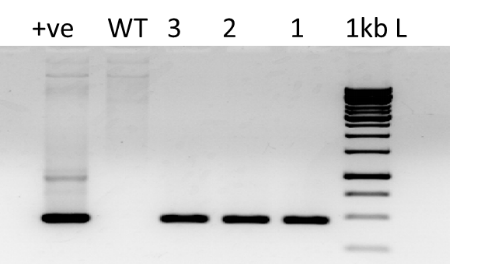


**Fig. S2**. PCR analysis of amplified transgene products from leaf tissues of the putative transgenic sweetpotato plants. Numbered lanes are transgenic plant lines, NT non-transformed plant for negative control, P vector for positive control and L 1-kb ladder (Thermo Fisher Scientific).


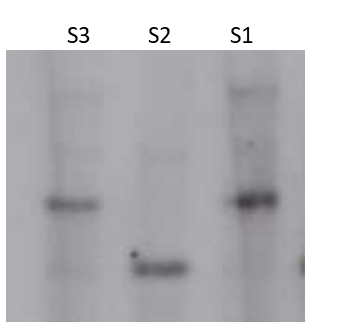


**Fig. S3.** Southern blot analysis of putative sweetpotato transgenics. 1. Southern blot showing integration of XvSap1 gene in transgenic plants. Digestion was performed using the *EcoRI* restriction enzyme. S1. 1, 2, and 3, are different XvSap1 transgenic sweetpotato lines.


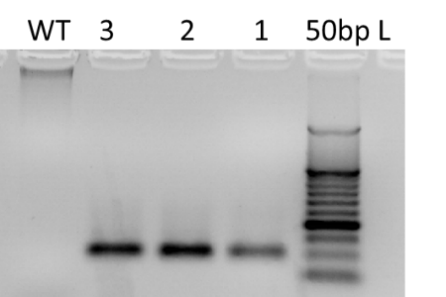


**Fig. S4** RT-PCR analysis confirming expression of *XvSap1* gene in leaves of transgenic sweetpotato plants. Numbered lanes are transgenic plant lines. *NT* non-transformed plant for negative control and *L* 50-bp ladder (New England Biolabs).

**Table S1 Primer sequences used for XvSap1gene amplification by PCR**

| **Primer** | **Sequence (5'- 3')** | **Product size (bp)** |
| --- | --- | --- |
| XvSap1-F | GCTAAACATGCGATCAAGCTCG | 464 |
| XvSap1-R1 | ATGATCCCGACGCTGTTTGAA |  |

**Table S2 Primer sequences used for synthesis of Southern blot probe**

| **Primer** | **Sequence (5'- 3')** | **Product size (bp)** |
| --- | --- | --- |
| cXvSap1-F | AATGAAGACCGACGTTGGAG | 413 |
| cXvSap1-R | CTCTGATTGTGTGGGCAATG |  |

**Table S3 Primer sequences used for cDNA amplification**

| **Gene** | **Primer** | **Sequence (5'- 3')** | **Product size (bp)** |
| --- | --- | --- | --- |
| XvSap1 | cXvSap1-F | AAGCTCTTCTTCCACCGACA | 103 |
|  | cXvSap1-R | CTCTGATTGTGTGGGCAATG |  |
| UBQ | cUBQ-F | CAAGCCGAAGAAGATCAAGC | 86 |
|  | cUBQ-R | GCACCTTTCCAGACTCATCC |  |

**Table S4. qRT-PCR analysis of XvSap1 transcript levels in leaves of transgenic sweetpotato XSP1, XSP2 and XSP3 transgenic lines during 12-day water deficit stress**

| No of days of drought stress | XSP1 | XSP2 | XSP3 |
| --- | --- | --- | --- |
| 0 | 2.6±1.9^a^ | 3±1.4^b^ | 1.7±1.0^c^ |
| 3 | 24.3±13.86^b^ | 20.93±19.76^c^ | 28.933±9.94^a^ |
| 6 | 132.83±70.67^a^ | 126.53±27.16^b^ | 113.583±16.25^c^ |
| 9 | 247.93±17.84^a^ | 197.93±27.38^b^ | 178.953±8.16^c^ |
| 12 | 162.93±53.52^a^ | 128.63±40.74^b^ | 142.613±55.17^b^ |

Means ± SD (Standard deviation) followed by different alphabets in each row are significantly different (P ≤0.05) using Fishers LSD.

**Table S5. Shoot length of wild type and *XvSap1* transgenic sweetpotato plants under normal and drought stress condition**

| **Growth condition** | **Wildtype** | **XSP1** | **XSP2** | **XSP3** |
| --- | --- | --- | --- | --- |
| Control | 11.98±0.38^a^ | 11.09±0.96^a^ | 10.63±1.12^a^ | 10.96±1.32^a^ |
| Drought | 5.38±0.58^b^ | 6.82± 0.68^a^ | 6.813±0.82^a^ | 6.65± 0.14^a^ |

Means ± SD (Standard Deviation) followed by different alphabets in each row are significantly different (P ≤0.05) using Fishers LSD.

**Table S6. Number of leaves of wild type and *XvSap1* transgenic sweetpotato plants under normal and drought stress conditions**

| **Growth condition** | **Wildtype** | **XSP1** | **XSP2** | **XSP3** |
| --- | --- | --- | --- | --- |
| Control | 8.67±1.53^b^ | 10.00±0.00^ab^ | 9.33± 0.58^ab^ | 10.67± 0.58^a^ |
| Drought | 4.67±0.58^b^ | 6.33±3 0.58^ab^ | 5.67±1.16^ab^ | 6.67± 1.16^a^ |

Means ± SD (Standard Deviation) followed by different alphabets in each row are significantly different (P ≤0.05) using Fishers LSD.

**Table S7. Yield fresh biomass of wild type and *XvSap1* transgenic sweetpotato plants under normal and drought stress conditions**

| **Growth condition** | **Wildtype** | **XSP1** | **XSP2** | **XSP3** |
| --- | --- | --- | --- | --- |
| Control | 485.79±16.95^a^ | 480.40± 26.60^a^ | 476.20± 32.0^a^ | 473.00±24.63^a^ |
| Drought | 160.74±14.25^b^ | 268.36±3.08^a^ | 261.42±5.67^a^ | 257.69±4.00^a^ |

Means ± SD (Standard Deviation) followed by different alphabets in each row are significantly different (P ≤0.05) using Fishers LSD.

**Table S8. Yield dry biomass of wild type and *XvSap1* transgenic sweetpotato plants under normal and drought stress conditions**

| **Growth condition** | **Wildtype** | **XSP1** | **XSP2** | **XSP3** |
| --- | --- | --- | --- | --- |
| Control | 266.50±33.90^d^ | 322.63±16.56^a^ | 318.70±20.8^b^ | 316.10±17.6^c^ |
| Drought | 147.74±13.09^d^ | 212.7±2.94^a^ | 205.58±5.68^b^ | 203.29±6.5^c^ |

Means ± SD (Standard Deviation) followed by different alphabets in each row are significantly different (P ≤0.05) using Fishers LSD.

**Table S9. Number of tubers of wild type and *XvSap1* transgenic sweetpotato plants under normal and drought stress conditions**

| **Growth condition** | **Wilt type** | **XSP1** | **XSP2** | **XSP3** |
| --- | --- | --- | --- | --- |
| Control | 3.67±0.58^a^ | 3.34±0.58^a^ | 3.67±1.53^a^ | 4.08± 1.10^a^ |
| Drought | 1.67±1.16^a^ | 3.67±1.16^b^ | 4.69±1.81^b^ | 4.33± 1.14 ^b^ |

Means ± SD (Standard Deviation) followed by different alphabets in each row are significantly different (P ≤0.05) using Fishers LSD.

**Table S10. Effect of drought on** **chlorophyll content of wild type and transgenic plants**

| **No. of days of drought stress** | **Wildtype** | **XSP1** | **XSP2** | **XSP3** |
| --- | --- | --- | --- | --- |
| -9 | 31.75±3.32^a^ | 32.04±2.75^a^ | 33.11±3.26^a^ | 30.74±4.97^a^ |
| -6 | 34.39±3.78^a^ | 33.89±3.15^a^ | 36.47± 1.53^a^ | 35.94±1.90^a^ |
| -3 | 38.54±2.69^a^ | 38.07±3.16^a^ | 41.05±1.80^a^ | 41.70±1.12^a^ |
| 0 | 42.32±2.29^a^ | 43.59±3.49^a^ | 44.41±1.45^a^ | 45.18±1.55^a^ |
| 3 | 42.24±0.68^b^ | 44.97±3.47^ab^ | 46.031±0.41a | 45.70±1.50^ab^ |
| 6 | 34.89±1.30^b^ | 46.43±3.93^a^ | 47.03±1.09^a^ | 45.85±1.48^a^ |
| 9 | 25.60±2.51^b^ | 35.98±1.160^a^ | 34.58±1.26^a^ | 33.45±2.63^a^ |
| 12 | 18.13±1.77^b^ | 25.07±1.10^a^ | 23.27±1.70^a^ | 22.70±2.41^a^ |

Means ± SD (Standard Deviation) followed by different alphabets in each column are significantly different (P ≤0.05) using Fishers LSD.

**Table S11. Proline content (µmol/g FW) of wild type and *XvSap1* transgenic sweetpotato plants under normal and drought stress conditions**

| **Growth condition** | **Wildtype** | **XSP1** | **XSP2** | **XSP3** |
| --- | --- | --- | --- | --- |
| Control | 4.27± 0.25^a^ | 3.99±0.31^a^ | 4.76± 0.32^a^ | 4.32±1.08^a^ |
| Drought | 16.36±1.543^b^ | 25.08±1.661^a^ | 26.41±2.89^a^ | 26.78±3.95^a^ |

Means ± SD (Standard Deviation) followed by different alphabets in each row are significantly different (P ≤0.05) using Fishers LSD.

**Table S12. MDA content (µmol/g FW) of wild type and *XvSap1* transgenic sweetpotato plants under normal and drought stress conditions**

| **Growth condition** | **Wildtype** | **XSP1** | **XSP2** | **XSP3** |
| --- | --- | --- | --- | --- |
| Control | 6.88±0.44^a^ | 6.46± 0.56^a^ | 5.72± 1.22^a^ | 5.57± 0.81^a^ |
| Drought | 23.63±3.30^a^ | 14.02±1.49^b^ | 15.50±3.07^b^ | 16.37±1.17^b^ |

Means ± SD (Standard Deviation) followed by different alphabets in each row are significantly different (P ≤0.05) using Fishers LSD.

**Table S13. Relative water content (%) of wild type and *XvSap1* transgenic sweetpotato plants under normal and drought stress conditions**

| **Growth condition** | **Wildtype** | **XSP1** | **XSP2** | **XSP3** |
| --- | --- | --- | --- | --- |
| Control | 82.26± 2.19^a^ | 82.03± 2.70^a^ | 79.01± 5.55^a^ | 80.4± 6.23^a^ |
| Drought | 35.61±1.61^b^ | 50.51± 3.24^a^ | 44.82± 6.1^8a^ | 47.00±6.18^a^ |

Means ± SD (Standard Deviation) followed by different alphabets in each row are significantly different (P ≤0.05) using Fishers LSD.
